# Ribosomal stress activates eEF2K–eEF2 pathway causing translation elongation inhibition and recruitment of Terminal Oligopyrimidine (TOP) mRNAs on polysomes

**DOI:** 10.1093/nar/gku996

**Published:** 2014-10-20

**Authors:** Angelo Gismondi, Sara Caldarola, Gaia Lisi, Giada Juli, Lidia Chellini, Valentina Iadevaia, Christopher G. Proud, Fabrizio Loreni

**Affiliations:** 1 Department of Biology, University Tor Vergata, Roma, 00133, Italy; 2 Centre for Biological Sciences, University of Southampton, Southampton, UK

## Abstract

The synthesis of adequate amounts of ribosomes is an essential task for the cell. It is therefore not surprising that regulatory circuits exist to organize the synthesis of ribosomal components. It has been shown that defect in ribosome biogenesis (ribosomal stress) induces apoptosis or cell cycle arrest through activation of the tumor suppressor p53. This mechanism is thought to be implicated in the pathophysiology of a group of genetic diseases such as Diamond Blackfan Anemia which are called ribosomopathies. We have identified an additional response to ribosomal stress that includes the activation of eukaryotic translation elongation factor 2 kinase with a consequent inhibition of translation elongation. This leads to a translational reprogramming in the cell that involves the structurally defined group of messengers called terminal oligopyrimidine (TOP) mRNAs which encode ribosomal proteins and translation factors. In fact, while general protein synthesis is decreased by the impairment of elongation, TOP mRNAs are recruited on polysomes causing a relative increase in the synthesis of TOP mRNA-encoded proteins compared to other proteins. Therefore, in response to ribosomal stress, there is a change in the translation pattern of the cell which may help restore a sufficient level of ribosomes.

## INTRODUCTION

The regulation of ribosome biogenesis is coordinated with cell growth and proliferation through mechanisms that have so far only been partially elucidated. For instance, rRNA synthesis is regulated through signaling pathways in response to growth stimuli (1). Moreover, it is now well-documented that signaling pathways regulate the translational activity of terminal oligopyrimidine (TOP) mRNAs that include all vertebrate ribosomal protein (RP) mRNAs. TOP mRNAs are relatively inefficiently translated and are therefore referred to as ‘weak’ mRNAs. External signals such as availability of growth factors, hormones and nutrients or stresses, can induce changes in the percentage of TOP mRNAs that is associated with polysomes from 25–45 to 60–75 and vice versa, respectively (2). Several lines of evidence indicate that PI3K and the mTORC1 complex are key modulators of TOP mRNAs translation after mitogenic stimulation (3). Rapamycin, which inhibits mTORC1 by binding to mTOR in a complex with the immunophilin FKBP12, has a variable effect on TOP mRNA translation. In HeLa cells, it completely blocks the recruitment of TOP messengers into polysomes following serum stimulation (2). In other cell lines, however, this inhibitory effect is only partial (3,4). The development of new, more efficient mTOR inhibitors as well as powerful high-throughput techniques to measure translational activity of mRNAs has recently allowed a further clarification of the relationship between mTORC1 and TOP mRNAs. Two laboratories reported that translation of TOP mRNAs is specifically dependent on mTORC1 activity (5,6). Moreover, the data of Thoreen *et al.* indicate that the mTORC1 substrate 4E-BP1 is a key player in their regulation (6), although this finding was not confirmed in a more recent publication (7).

An important issue that remains to be explored is the operation of regulatory mechanisms to coordinate the synthesis among the many ribosomal components. It has been shown that RPs are normally produced in larger amounts than are needed for ribosome production, and the excess proteins are then degraded in the nucleolus (8). Therefore, it is possible that coordination among ribosomal components is achieved mainly by degradation of excess molecules. More recently, a number of reports have suggested that perturbations of ribosome biogenesis due to a variety of causes (ribosomal stress) can activate a specific checkpoint and block cell proliferation mostly through a p53-dependent mechanism (9–15). This occurs, for example, in the case of conditional deletion of RPS6 (16,17) or in response to drugs which disrupt nucleolar structures (13,18). Interestingly, Fumagalli *et al.* reported that impairment in the synthesis of an RP induces a regulatory response that affects the synthesis of other RPs (14). In fact, the authors observed that following depletion of an RP of the small subunit (RPS6, RPS7 or RPS23), TOP mRNAs are recruited onto polysomes and, as a consequence, presumably more actively translated.

Here, we have studied the regulatory processes which are activated in response to defect in the synthesis of ribosomal components. We find that the deficiency of RPS19 or other RPs causes a slowdown of translation elongation. As a consequence, there is an increase of the percentage of TOP mRNAs associated with polysomes. The result of this response is that the synthesis of proteins encoded by TOP mRNAs (which include all RPs) is maintained relative to the production of other proteins, which is inhibited. This allows for continued RP synthesis for new ribosome production.

## MATERIALS AND METHODS

### Cell culture and transient transfection

K562C and TF-1C (human erythroleukemia) cells were maintained in RPMI 1640 medium. PC3 (human prostate carcinoma) cells were maintained in Dulbecco's modified Eagle Medium. All media were supplemented with 10% fetal calf serum, 50 units/ml penicillin and 50 mg/ml streptomycin. TF-1C medium was also supplemented with 5 ng/ml Granulocyte-Macrophage Colony-Stimulating Factor. Cells were incubated at 37°C in a humidified atmosphere with 5% CO_2_. TF-1C and K562C cells, expressing inducible siRNA targeting RPS19 mRNA, were prepared in Karlsson's laboratory (19). Expression of siRNA was induced by adding 2 μg/ml of doxycycline for four days. PC3 cells (5 × 10^6^) were transiently transfected with 100 nM siRNA and 10 μl of Interferin transfection reagent (Polyplus transfection) according to the manufacturer's protocol. After 48 h, they were harvested and analyzed by polysomal gradient assay or by western blot. The siRNA target sequences were as follows: sense 5′-UAUUUAAGGGCUUUCUUAC-3′ for human RPS6, sense 5′-GAUGGCAGCCGGCUCAUAA-3′ for human RPS7, sense 5′-GAGAUCUGGACAGAAUCGC-3′ for RPS19, sense 5′-GGUGCGGGAGUAUGAGUUA-3′ for RPL11 and sense- 5′-GACACGCGACUUGUACCAC-3′ for control siRNA (siCNT).

K562C cells were treated with 50 ng/ml cycloheximide (Sigma-Aldrich) for 1 h and PC3 cells with 25 nM actinomycin D (Sigma-Aldrich) for 12 h. Both K562C and PC3 cells were treated with 1 μM PP242 (Sigma-Aldrich) for 2 h.

### Cap-affinity chromatography

For the isolation of eIF4E (cap)-associated proteins, K562C and PC3 cells were lysed in buffer (containing 50 mM Hepes, 75 mM NaCl, 10 mM MgCl_2_, 1 mM DTT, 8 mM EGTA, 10 mM β-glycerophosphate, 0.5 mM Na_3_VO_4_, 0.5% Triton-X-100 and protease inhibitor cocktail). Cell extracts were incubated for 10 min on ice and centrifuged at 16 000 g for 10 min at 4°C. The supernatant was collected, protein was quantified by the Bradford assay and 0.5 mg of protein extracts was diluted in 0.5 ml of lysis buffer and incubated with 10 μl of m^7^GTP-sepharose (GE Healthcare) plus 10 μl of Sepharose CL-4B (GE Healthcare) at 4°C for 90 min under constant shaking. After centrifugation for 30 s at 2500 g, beads were washed three times with lysis buffer, resuspended in Laemmli buffer and subjected to western blot analysis. Blots were decorated with antibodies against eIF4E (Cell Signaling), eIF4G (Cell Signaling), 4E-BP1, RPS19 (monoclonal (20)) or rabbit polyclonal β-tubulin (Santa Cruz).

### Protein analysis

To prepare protein total extracts, cells were washed in phosphate-buffered saline (PBS) and lysed in high-salt lysis buffer (50 mM Tris-HCl pH 7.5, 350 mM NaCl, 1 mM MgCl_2_, 0.5 mM EDTA, 0.1 mM EGTA, 1% Nonidet P-40, 1 mg/ml aprotinin, 1 mg/ml leupeptin, 1 mg/ml pepstatinA, 100 mg/ml PMSF). After 30 min on ice, nuclei and other organelles were collected by centrifugation at 16 000 g for 15 min. The supernatant was transferred into a new tube and protein concentrations were determined using the Bradford assay.

Proteins were separated on sodium dodecyl sulfate (SDS) polyacrylamide gels containing 12% acrylamide, transferred onto nitrocellulose Protran membrane (Schleicher and Schuell) and incubated with the following primary antibodies: rabbit monoclonal antibody specific for p70S6K1 (Upstate), rabbit polyclonal for phospho-p70S6K1 specific for threonine 389 (Santa Cruz), 4E-BP1 (kindly provided by Nahum Sonenberg, Montréal, Canada), mouse monoclonal anti-RPS19 (20), rabbit polyclonal specific for β-tubulin (Santa Cruz), rabbit anti-β-actin (Sigma-Aldrich), rabbit polyclonal anti-eEF2 (Cell Signaling), rabbit polyclonal anti-phospho-eEF2 antibody specific for threonine 56 (custom made by Eurogentec). Primary antibodies were revealed using horseradish peroxidase-conjugated goat anti-rabbit or anti-mouse Ab (Jackson Immunoresearch) and the ECL chemiluminescence detection system (Pierce). Quantification analyses were performed by LAS3000 Image System (Fuji) and ImageQuant software (GE Healthcare).

### Protein labeling

For general protein synthesis, 2 × 10^5^ K562C or PC3 cells were incubated for 30 min with [^35^S] methionine/cysteine (PRO-MIX, GE Healthcare, >1000 Ci/mmol) to a final concentration of 10 μCi/ml. Cells were lysed in PBS–SDS buffer (150 mM NaCl, 2.7 mM KCl, 8 mM Na_2_HPO_4_, 1.4 mM KH_2_PO_4_ and 0.1% SDS) and proteins were precipitated in 10% (w/v) trichloroacetic acid (TCA). After three washes with 5% (w/v) cold TCA, the insoluble material was collected on GFC filters (Whatman) and the incorporated radioactivity was measured in scintillation counting.

For immunoprecipitation, PC3 cells (4 × 10^6^) or K562C (15 × 10^6^) were incubated in 2.5 or 5 ml of methionine- and cysteine-free medium supplemented with 10% dialyzed FBS and containing [^35^S]methionine/cysteine (PRO-MIX, GE Healthcare, >1000 Ci/mmol) to a final concentration of 300 μCi/ml for 30 min at 37°C. The labeling medium was removed, and the cells were washed once in PBS, frozen in liquid nitrogen and stored at −70°C to be analyzed later or immediately processed.

### Immunoprecipitation

Radiolabeled PC3 or K562C cells were lysed in buffer with detergent (25 mM HEPES pH 7.6, 100 mM NaCl, 0.5% Nonidet P-40, 0.1 mM EDTA, 10% glycerol, 1 mM DTT, protease inhibitor cocktail). Lysates were incubated with rotation at 4°C for 10 min and nuclei and other organelles were removed by centrifugation at 13 000 g for 15 min at 4 °C. Equal amounts of protein (1–2 mg) were precleared by incubation with 15–30 μl of magnetic beads (Dynabeads Protein G -Millipore) for 45 min at 4°C. Subsequently, the supernatant was incubated with 8 μg of anti-eEF1A (Millipore) or 4 μg of anti-SOD1 (Millipore) overnight at 4°C under agitation. The extracts were then incubated for 60 min with 50 μl of Dynabeads Protein G. The immunocomplexes were isolated by a magnetic support and washed five times with 500 μl of lysis buffer. Proteins bound to the beads were eluted in SDS-PAGE sample buffer and heated at 90°C for 10 min. Immunoprecipitated proteins were separated on 4–12% NuPAGE Bis-Tris gel (Invitrogen) and electroblotted onto nitrocellulose (Protran; Schleicher and Schuell). Newly synthesized proteins were detected by exposing the membrane to a Phosphorimager screen at room temperature for 16 h; while total protein was detected by incubating with antibody against EF1A or SOD. Band intensities were quantiﬁed by densitometry using with the ImageQuant software (GE Healthcare).

### Polysomal RNA analysis

K562C, TF-1C and PC3 cells (1–2 × 10^6^) were washed once with PBS buffer (150 mM NaCl, 2.7 mM KCl, 8 mM NaH_2_PO_4_ and 1.4 mM K_2_PO_4_), lysed with 300 μl of lysis buffer (10 mM NaCl, 10 mM MgCl_2_, 10 mM Tris-HCl pH 7.5, 1% triton-X100, 1% sodium deoxycholate, 36 U/ml RNase inhibitor (Promega), 1 mM dithiothreitol) and transferred into a microcentrifuge tube. After 2 min of centrifugation at 16 000 g at 4°C, the supernatant was frozen in liquid nitrogen and stored at −70°C to be analyzed later, or immediately layered onto a 15–50% (w/v) sucrose gradient (containing 30 mM Tris-HCl pH 7.5, 100 mM NaCl and 10 mM MgCl_2_) and centrifuged in a Beckman SW41 rotor for 110 min at 170 000 g. Fractions were collected while monitoring the optical density at 254 nm. RNA was extracted from each polysomal fraction, from cells and from the only cytoplasmic compartment by the proteinase K method. For northern analysis, RNA was fractionated on formaldehyde-agarose gels and transferred to GeneScreen Plus membrane (PerkinElmer Life Sciences). Northern blotting was performed essentially as recommended by the manufacturer. Radioactive probes were prepared by the random primer technique using DNA fragments isolated from plasmids containing PCR-amplified cDNA sequences. Quantitation of northern blot filters was done with a PhosphorImager (GE Healthcare).

### Ribosome transit time mesaurements

K562C cells (3 × 10^7)^ were suspended in 5 ml of labeling medium (RPMI 1640 supplemented with 10% dialyzed bovine calf serum, 3.5 g glucose/l and 2 mM glutamine) for 20 min and then 12 μCi/ml [^35^S]methionine/cysteine cell labeling mix (PRO-MIX) was added. At the times indicated, cells were harvested, pelleted and resuspended in 0.5 ml of RSB (10 mM NaCl, 10 mM Tris-HCl at pH 7.4, 15 mM MgCl_2_ and 100 μg/ml heparin). Cells were lysed by adding 70 μl of lysis buffer (10% Triton X-100, 10% deoxycholate), rapidly mixed and incubated few min in ice. Nuclei and mitochondria were pelleted by centrifugation for 10 min at maximum speed in a microfuge at 4°C. Five hundred microliter of the PMS (post-mitochondrial supernatant) was mixed with an equal volume of polysomal buffer (25 mM Tris-HCl at pH 7.4, 10 mM MgCl_2_, 25 mM NaCl, 0.05% Triton X-100, 0.14 M sucrose, 500 μg/ml heparin), and 450 μl was removed to measure incorporation of [^35^S]-methionine and -cysteine into total protein (nascent and completed). Polysomes were pelleted by centrifugation of the remaining supernatant at 100 000 g for 20 min at 4°C in a Beckman 70.1Ti rotor. Four hundred and fifty microliter of post-ribosomal supernatant (PRS) was removed to measure the incorporation of [^35^S]-methionine/cysteine into completed proteins. PMS and PRS samples were mixed with equal volumes of 20% TCA containing 10 mM each of methionine and cysteine, placed in ice for 20 min and then TCA precipitates were collected on a glass fiber filter (GF/C; Whatman). Filters were washed with ice-cold 10% TCA, rinsed once with ethanol and air dried before being subjected to liquid scintillation counting.

## RESULTS

### Ribosomal stress causes recruitment of TOP mRNA on polysomes

**Figure 1.**
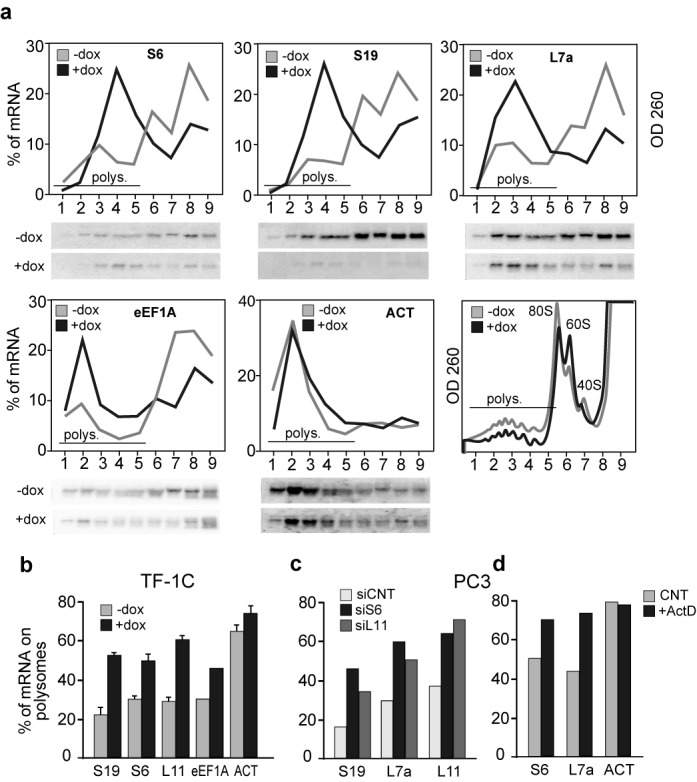
Polysomal association of TOP mRNAs during ribosomal stress. (**a**) Cytoplasmic extracts from K562C cells untreated (-dox) or treated for four days with doxycycline (+dox) were separated on sucrose gradients. Fractions were collected and polysomal profiles were obtained while monitoring the optical density at 260 nm (last panel). RNA extracted from each fraction was analyzed on northern blots with probes for the TOP mRNAs RPS6 (S6), RPS19 (S19), RPL7a (L7a) and eIF1A or β-actin (as a non-TOP control). Quantification of the signals is reported as a linear plot of the percentage of mRNA in each fraction. (**b**) Experiments performed in TF-1C cells as in (a). Quantification is shown as a bar graph of the percentage of messenger (mRNA) associated with polysomes (fractions 1–5). Values represent the mean ± S.E. of at least three independent experiments. (**c**) Cytoplasmic extracts from PC3 cells transfected with a control siRNA (siCNT) or siRNAs against RPS6 (siS6) or RPL11 (siL11) were analyzed as in (a) with probe specific for the TOP mRNA RPS19 (S19), RPL7a (L7a) and RPL11 (L11). (**d**) Cytoplasmic extracts from PC3 cells treated for 12 h with low doses (25 nM) of actinomycin D (ActD) were analyzed as in (c).

To investigate the translational efficiency of TOP mRNAs in response to the depletion of RPS19, we used two modified cell lines, K562C and TF-1C, both infected with a lentiviral vector which allows doxycycline (dox)-inducible expression of siRNA specific for the RPS19 mRNA (19). To assess the amount of TOP mRNA associated with polysomes during the induction of RPS19 deficiency, cytoplasmic extracts from K562C and TF-1C cells (untreated or treated for four days with dox) were separated on sucrose gradients. Fractions were collected and analyzed by northern blot. Consistent with the findings of other laboratories (14,21), we observed an increase of polysomal association of the TOP mRNAs RPS19, RPS6, RPL7a, eEF1A (in K562C and TF-1C), RPL11 (only in TF-1C), in RPS19-depleted cells (Figure 1a and b). To rule out an effect of dox itself, we used a K562 cell line infected with a lentiviral vector inducible for the expression of control siRNA (iSCR). In this case, dox treatment did not cause any change in the polysomal association of TOP mRNAs (Supplementary Figure S1a). As a further control, to verify that RPS19 depletion did not alter TOP mRNA turnover or subcellular localization, we analyzed total, nuclear and cytoplasmic mRNA levels in control and dox-induced K562C cells. The results confirmed that TOP mRNA level did not change following RPS19 depletion (Supplementary Figure S1b).

To test if TOP mRNA recruitment could be observed in other cell types after depletion of other RPs, we used the prostate cancer cell line PC3. In this case we carried out transient transfection of specific siRNAs to induce the depletion of RPS6 (siRPS6), RPL11 (siRPL11) and an unrelated siRNA as a control (siCNT). The association of selected TOP mRNAs with polysomes was analyzed as described above, and the results, reported as column graphs in Figure 1c, indicate that depletion of RPS6 or RPL11 causes an increase of the percentage of TOP mRNAs (RPS19, RPL7a, RPL11) associated with polysomes.

Finally, to analyze the effect of ribosomal stress caused by another mechanism, we treated PC3 cells with low doses of actinomycin D (actD), which is known to inhibit rRNA transcription. Analysis of polysomal association of TOP mRNAs, shown in Figure 1d, indicates that actD treatment increases the levels of the RPS6 and RPL7a mRNAs which are associated with polysomes.

### Ribosomal stress does not affect mTORC1 and ERK signaling but inhibits general protein synthesis

**Figure 2.**
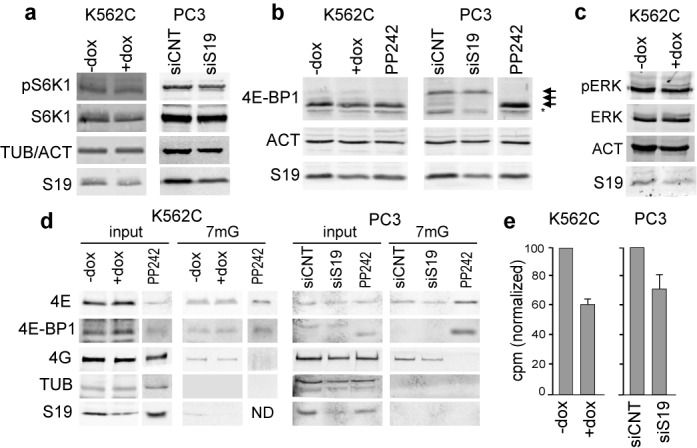
Analysis of mTORC1 pathway and protein synthesis. (**a**) Protein extracts, prepared from K562C cells treated with or without dox and from PC3 cells transfected with an unrelated siRNA (siCNT) or with a siRNA against RPS19 (siS19), were analyzed by western blot with the indicated antibodies. (**b**) The same extracts plus samples from cells treated with the mTOR kinase inhibitor PP242 (1μM) were analyzed to distinguish different phosphorylated forms of 4E-BP1. Small arrows indicate the different phosphorylation forms of 4E-BP1 and an unspecific signal is indicated by asterisk. PP242 lane of PC3 extracts is from the same gel. (**c**) Same as in (a). (**d**) K562C and PC3 cells treated as in (a) were subjected to cap-column pull-down assay using 7-methyl-GTP Sepharose beads. Cell lysates (input) or proteins bound to m^7^GTP-Sepharose (m^7^G) were analyzed by immunoblotting using indicated antibodies. PP242 lane is from a different gel. (**e**) K562C and PC3 cells treated as in (a) were incubated with [^35^S]-met/cys mix for 30 min. [^35^S]-amino acid incorporation was measured by scintillation counting and the cpm (counts per minute) obtained were normalized to the total amounts of protein. Results are shown as a bar graph considering control cells as 100% and represent the mean ± S.E. of three independent experiments.

To assess possible changes in known signaling pathways, we analyzed the phosphorylation status of the mTORC1 downstream targets S6K1 and 4E-BP1. As a control, we treated cells with the mTOR inhibitor PP242 which is known to abolish 4E-BP1 phosphorylation. As shown in Figure 2a and b, western analysis indicated that RPS19 depletion in K562C or PC3 cells did not increase the phosphorylation of S6K or 4E-BP1. We also analyzed the phosphorylation status of ERK kinase observing no alteration caused by RPS19 depletion in K562 cells (Figure 2c). As an additional analysis of the translation efficiency in RPS19-depleted cells, we monitored the assembly of the eEF4F initiation complex by pull-down assays with 7-methyl-GTP-Sepharose beads (Figure 2d). Consistent with the data on 4E-BP1 phosphorylation, we observed that RPS19 depletion did not change the relative amounts of eIF4G and 4E-BP1 associated with eIF4E (and therefore retained on the beads). These data are consistent with a report by Zinzalla *et al.* that observed no effect of ribosomal stress on mTORC1 after depletion of RPL7 in HeLa cells (22). However, it should be mentioned that a mild activation of mTORC1 was observed in zebrafish after depletion of RPS14 or RPS19 (23).

To further analyze the effect of RPS19 depletion, we measured general protein synthesis. For this purpose K562C cells, untreated or treated with dox, or PC3 cells transfected with siRNA specific for RPS19, were labeled for 30 min with a mixture of [^35^S]methionine/[^35^S]cysteine. The amount of [^35^S] incorporated into newly synthesized proteins, reported as a column graph in Figure 2e, indicates that RPS19 depletion causes a reduction of general protein synthesis both in K562C cells (down to 60% of control), and in siRNA-treated PC3 cells (70% of control). Thus, as anticipated, interfering with ribosome biogenesis does indeed impair overall protein synthesis. Although depletion of an RP causes a decrease in the amount of ribosomes in the cell, we think that this is not the direct cause of the reduction of protein synthesis. In fact, the analysis of absorbance profiles of RP-depleted cells shows a decrease of ribosomal subunits (40S or 60S according to the depleted RP) and 80S particles whereas the amount of polysomes does not change (Supplementary Figure S2a and b). To gain a more quantitative measure of this observation, we used a probe specific for 18S rRNA to evaluate the amount of ribosomes present in the different fractions of a sucrose gradient. The results indicate that in RPS19-depleted PC3 cells, total 18S rRNA per microgram of total protein is reduced to ∼70% and this is similar to the decrease of RPS19 measured on western blot (Supplementary Figure S2c). However, the amount of 18S rRNA in the polysomal fractions shows only a minimal reduction (5% decrease) indicating that the amount of ribosomes engaged in protein synthesis does not change (Supplementary Figure S2c).

### Protein synthesis is inhibited at the elongation level

**Figure 3.**
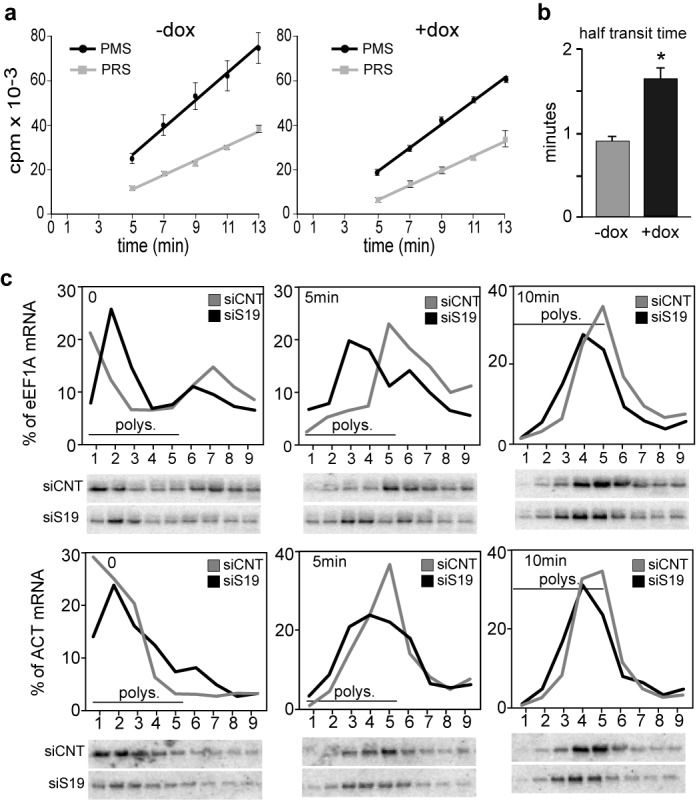
Analysis of translation elongation. (**a**) Ribosome transit time measurements in K562C cells, either untreated (-dox) or treated for four days with dox (+dox), were determined by measuring the kinetics of [^35^S]-methionine/[^35^S]-cysteine incorporation into total protein in PMS and PRS. The radioactivity at each time point is presented as a mean ± S.E. of three independent experiments. The transit time was obtained from the displacement in time between the intercepts of the two lines on the time axis, which were determined by linear regression analysis. (**b**) Means ± S.E. of three independent experiments. * Student t-test *P* < 0.05. (**c**) Cytoplasmic extracts from PC3 cells transfected with a control siRNA (siCNT) or with siRNA against RPS19 (siS19) untreated (*T* = 0’) or treated for 5 and 10 min with harringtonine (*T* = 5’, *T* = 10’) were separated on sucrose gradients and analyzed as in Figure 1.

Absorbance profiles of RP-depled cells (Figure 1) did not show a decrease in the average polysome size suggesting that initiation was not impaired. Therefore to explain the decrease in protein synthesis, we examined the translation elongation rate by analyzing the ribosome transit time (24). This was performed by measuring the kinetics of radioactive amino acid incorporation into total protein in PMS and into completed polypeptides released from ribosomes into PRS (Figure 3a). The average half-transit time was determined from the displacement in time between the two lines corresponding to the PMS and PRS data plotted as a function of time (explained in Supplementary Figure S3), and was calculated to be 53 and 96 s, for control and dox-treated K562C cells, respectively. These data, shown in the column graph of Figure 3b, indicate that RPS19 depletion slows elongation by ∼50%. The fact that the lines of the plots are not parallel probably reflects incomplete recovery of released labeled polypeptides (PRS) in the supernatant, likely because some of the new polypeptides are associated with entities that are in the pellet (e.g. ribosomes, organelles). Similar profiles are shown in a number of published reports (25,26). Although the non-parallel nature of the plot might affect the precision of the measurements, it does not change the overall conclusion that elongation rates are altered.

To study further the elongation rates using a different method, we analyzed the kinetics of ribosome run-off from polysomes after inhibition of translation initiation. For this purpose PC3 cells, transfected with control or RPS19-specific siRNA, were treated with 2 μg/ml of harringtonine, which blocks initiation by inhibiting the first round of elongation (27), and polysome analyses were performed at different times. As shown in Figure 3c and Supplementary Figure S4, the TOP mRNAs for eEF1A (28), RPL4 and RPS7 shift gradually out of polysomes more slowly in RPS19-depleted (siRPS19) cells compared to the control (siCNT). The same analysis on the control β-actin mRNA showed a similar behavior (Figure 3c and Supplementary Figure S4). This confirms (i) that elongation rates are indeed slower in RPS19-depleted cells and (ii) that such rates are likely similar for TOP and non-TOP mRNAs. This effect on elongation is rather unexpected, as deficiency of a ribosomal subunit would be expected to affect initiation. We think that this is a specific mechanism to maintain homeostatic equilibrium in ribosome synthesis (see discussion).

### Pharmacological inhibition of elongation causes a recruitment of TOP mRNA on polysomes

**Figure 4.**
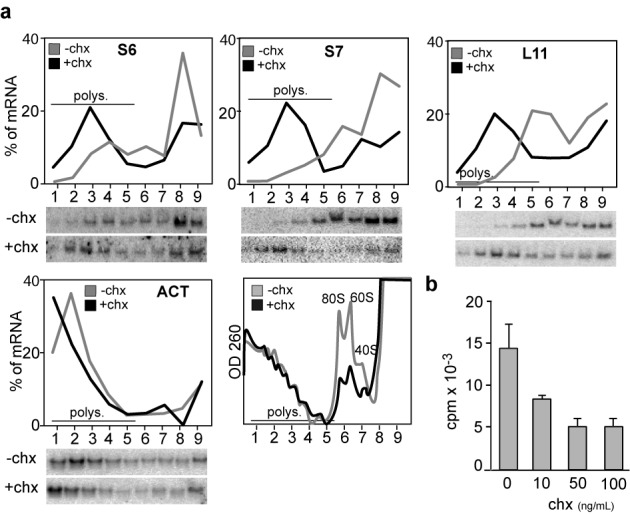
Polysomal association of TOP mRNAs during cycloheximide treatment. (**a**) Cytoplasmic extracts from K562C cells untreated (-chx) or treated (+chx) with cycloheximide (50 ng/ml) for 1 h were separated on sucrose gradients and analyzed as in Figure 1. (**b**) K562C cells treated with different concentration of cycloheximide (chx) were incubated with [^35^S]-met/cys mix for 30 min. [^35^S] incorporation was measured by scintillation counting and results are shown as a bar graph of the means ± S.E. of three experiments.

It has long been known that partial inhibition of translation elongation can actually increase the polysomal association of mRNAs that exhibit a low intrinsic initiation efficiency (29). The relatively poor initiation efficiency of TOP mRNAs, compared to other housekeeping mRNAs, was noted in the initial molecular analyses (30,31). In fact, the percentage of TOP mRNA associated with polysomes ranges from 25 to 70 in different cell types and growth conditions, whereas the polysomal proportion of other housekeeping mRNAs (e.g. actin) is always higher and varies from 70% to >90% (2,31,32). To address the effect of elongation inhibition on the polysomal association of TOP mRNAs, we treated K562C cells with low doses (50 ng/ml) of the elongation inhibitor cycloheximide for 1 h. This treatment causes an inhibition of [^35^S]met/cys incorporation to ∼35% of controls (Figure 4b) and induces a recruitment of free ribosomal subunits into polysomes (Figure 4a, last panel). We measured the amount of RPS6, RPS7, RPL11 and β-actin mRNAs associated with polysomes as above. The results are shown as a linear graph of the percentage of mRNA in the different fractions (Figure 4a). Consistent with the hypothesis that slowing elongation augments the polysomal association of poorly initiating (‘weak’) mRNAs, cycloheximide treatment caused a recruitment of TOP mRNAs into polysomes. The effect is evident for the ‘weak’ TOP mRNAs (from 20–40% to 60–70% on polysome) but is barely observed for the ‘strong’ β-actin mRNA.

### The synthesis of the TOP mRNA-encoded eIF1A does not change during ribosomal stress

**Figure 5.**
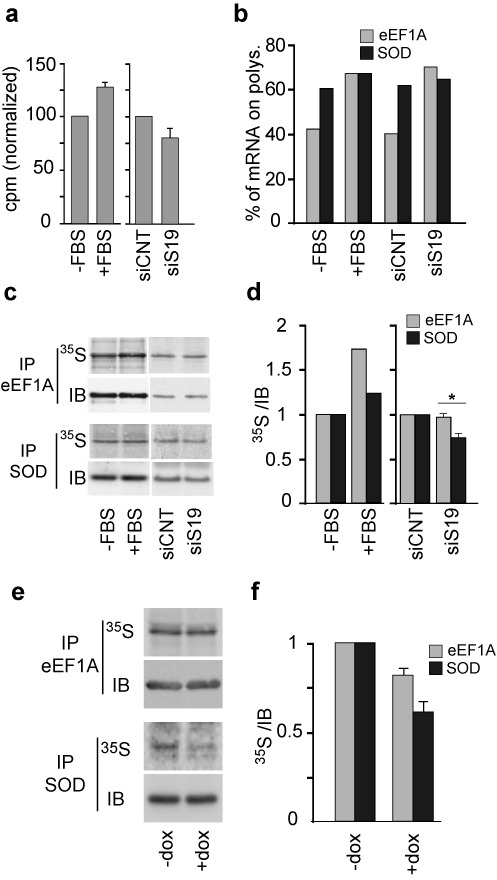
Analysis of newly synthesized eEF1A and SOD. (**a**) PC3 cells untransfected (−FBS, +FBS), transfected with a control siRNA (siCNT) or with siRNA against RPS19 (siS19), were grown without serum for 16 h. An aliquot of untransfected cells was stimulated with serum for 1 h (+FBS) prior to analysis. Cells were radiolabeled with [^35^S]met/[^35^S]cys mix for 30 min and analyzed by scintillation counting. Values (cpm) were normalized to protein amount and the means ± S.E. of three independent experiments are shown as a bar graph considering −FBS and siCNT cells as 100. (**b**) Portions of the samples described in (a) were subjected to polysome association analysis as described in Figure 1. The percentages of eEF1A and SOD mRNAs associated with polysomes are reported as a bar graph. (**c**) Part of the samples described in (a) (equal amount of proteins) was immunoprecipitated with antibodies against eEF1A or SOD, separated on SDS-PAGE and subjected to western blot. Radiolabeled eEF1A and SOD were detected by direct exposure of the membrane to phosphor screens (^35^S). Total eEF1A and SOD in immunoprecipitates (IP) were detected with specific antibodies (IB). (**d**) Quantification of radiolabeled/total protein ratios ([^35^S]/immunoblot; IB) of experiment c. Ratios of siS19 cells are the mean ± S.E. of at least three gels from two independent experiments. Student's t test (*P = 0.01). (**e**) K562C cells +/− dox were analyzed as in (c). (**f**) Quantitative analysis of (e), means of three experiments ± S.E.

The observed increase in the polysomal association of TOP mRNAs in response to ribosomal stress is, therefore, consistent with an inhibition of translation elongation favoring the accumulation of ribosomes on weak mRNAs. Importantly, this effect could favor the synthesis of, e.g. RPs, relative to general protein synthesis in the face of inadequate RP availability, in effect helping to maintain ribosome production. To assess this idea, we induced RPS19 depletion in PC3 cells and, after 30 min labeling with a [^35^S]Met/Cys, we analyzed: (i) overall protein synthesis rates (Figure 5a); (ii) the percentages of eEF1A mRNA (example of a TOP mRNA) and SOD mRNA (non-TOP) associated with polysomes (Figure 5b) and (iii) quantitation of newly synthesized eEF1A and SOD by specific immunoprecipitation followed by quantitation of the associated radiolabel (Figure 5c and d). For this purpose, the immunoprecipitated complexes were separated on SDS-PAGE and transferred onto nitrocellulose membranes. We then measured both radiolabeled protein (by direct exposure of the membrane) and total protein (by immunoblot quantitation; Figure 5c). The results are reported graphically as the ratio of [^35^S]labeled:total protein (Figure 5d). eEF1A was selected because it is encoded by a well-established TOP mRNA and studying eEF1A obviated various problems encountered in attempts to immunoprecipitate ribosomes. In this experiment, PC3 cells (untransfected, transfected with control siRNA, or with siRNA specific for RPS19) were serum-starved for ∼16 h and then an aliquot of untransfected cells was stimulated with serum for 1 h. Serum stimulation after starvation was used as a control since it is known to promote polysomal loading of TOP mRNAs mainly as a consequence of regulation of initiation of their translation (2).

The results indicate that: (i) serum stimulation caused a modest upregulation of general protein synthesis whereas RPS19 depletion induced a slight decrease (Figure 5a). (ii) As expected from earlier studies (28,33), the percentage of the eEF1A mRNA associated with polysomes increased after serum stimulation. A similar increase occurs following depletion of RPS19, as also shown in Figure 1. In contrast, the polysomal association of the SOD mRNA was barely affected either by serum stimulation or RPS19 depletion. (iii) Notably, the ratio [^35^S]-labeled:total eEF1A increases during serum stimulation and is not altered by RPS19 depletion, whereas the ratio [^35^S]:total SOD increases slightly during serum stimulation and reproducibly decreases during RPS19 depletion. Similar results were obtained in K562C cells where, when RPS19 was depleted, the ratio [^35^S]:total SOD showed a more evident decrease compared to the [^35^S]:total eEF1A ratio (Figure 5e and f). These data show that the production of eEF1A (encoded by a prototypical TOP mRNA) is preserved during ribosomal stress relative to the synthesis of SOD (not coded by a TOP mRNA), which is impaired.

### RP depletion induces activation of eEF2K and eEF2 phosphorylation

**Figure 6.**
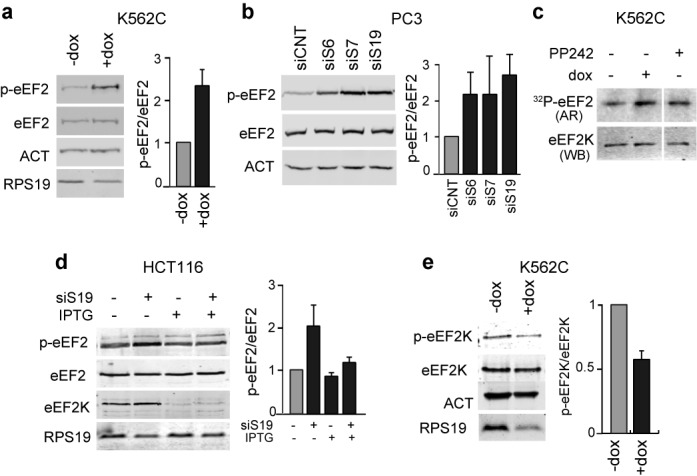
Analysis eEF2 and eEF2K during ribosomal stress. (**a**) Protein extracts from K562C cells were analyzed as in Figure 2a with the indicated primary antibodies. Phospho-eEF2/eEF2 ratio is reported on the right with untreated sample set as 1 (mean ± S.E. of at least three independent experiments). (**b**) PC3 cells were transfected with a control siRNA (siCNT) or with siRNAs against RPS6 (siS6), RPS7 (siS7) and RPS19 (siS19). Proteins were analyzed as in (a). (**c**) Protein extracts from K562C cells +/− dox were incubated with recombinant eEF2, [γ-^32^P]ATP and Ca^2+^/CaM. Samples were then analyzed by SDS-PAGE and autoradiography (AR) or western blot (WB). Extracts treated with PP242 were used as control. The space between lanes denotes that these are non-adjacent lanes from the same gel. (**d**) HCT116 cells treated with IPTG 1 mM to induce eEF2K knock-down were transfected with siRNA against RPS19 (siS19). Proteins were analyzed by western blot with the indicated antibodies. Quantification is reported on the right as in (a). (**e**) Protein extracts from K562C cells were analyzed as in (a) with the indicated primary antibodies. The phospho-eEF2K/eEF2K ratio is reported on the right with untreated sample as 1 (mean ± S.E. of at least three independent experiments).

The phosphorylation of eEF2 inhibits translation elongation (34). Thus, to examine the mechanism by which elongation is inhibited in RPS19-depleted cells, we analyzed the phosphorylation status of eEF2 in K562C and PC3 cells. The results (Figure 6a and b) indicate that, consistent with an inhibition of elongation, the level of phosphorylated eEF2 (Thr56) increases by ∼2.5-fold in RPS19-depleted K562C cells and in PC3 cells after depletion of RPS6, RPS7 or RPS19. The enzyme that phosphorylates eEF2 on the regulatory site (Thr56) is eEF2 kinase (eEF2K). We tested the activity of eEF2K in K562 cells following induction of RPS19 depletion. We performed an *in vitro* kinase assay using dox-treated K562C cell extracts using eEF2 as substrate. The data (Figure 6c) show that eEF2K is more active following RPS19 knock-down suggesting it is its activation that leads to the increased phosphorylation of eEF2.

Then, we used an HCT116 cell line in which depletion of eEF2K can be induced by IPTG treatment. We observed that inhibition of expression of eEF2K abolished the increase of phospho-eEF2 induced by RPS19 depletion (Figure 6d), indicating that eEF2K is indeed responsible for the phosphorylation of eEF2 in this setting.

Finally, it has been shown that eEF2K is inhibited by phosphorylation on Ser366 (35). To further address the activity of the kinase during ribosomal stress, we used antibodies specific for the phosphorylated form (Ser 366) of the protein. Western analysis, reported in Figure 6e, shows that RPS19 depletion in K562 cells causes decreased phosphorylation of eEF2K on Ser366, consistent with the observed enhancement its kinase activity.

## DISCUSSION

We report here a previously uncharacterized response to defects in ribosome biosynthesis that involves translational reprogramming dependent on inhibition of translation elongation. We find that depletion of an RP, or administration of low doses of actinomycin D, induces an increase in the proportion of TOP mRNAs that are associated with polysomes. This can be observed both in p53-positive (14) and p53-negative cells (our data), suggesting that this tumor suppressor does not play a role in the phenomenon. It has been known for a long time that the polysomal association of TOP mRNA correlates with the growth status of the cell (36). The recruitment of TOP mRNAs onto polysomes in response to (ribosomal) stress was therefore unexpected, considering that it was occurring in parallel to protein synthesis inhibition and impaired cell growth. We hypothesized that this could be the consequence of a mechanism for achieving homeostasis in ribosome biosynthesis, whereby a defect in ribosome synthesis induces a compensatory increase of RP synthesis. A similar regulation has been already observed during the development of the Xenopus ‘anucleolate’ mutant (37) and in cultured Xenopus cells treated with transcriptional inhibitors (38). However, the mechanism underlying those responses was not explored.

For this reason, we started to address the response to ribosomal stress by measuring the activity of the mTORC1 pathway since it is implicated in the translational regulation of TOP mRNAs (reviewed in (36)). Analysis of the phosphorylation of the direct mTORC1 targets S6K1 and 4E-BP1 indicated that the activity of mTORC1, at least in our cellular models (K562C and PC3 cells), was not affected by ribosomal stress. In addition, general protein synthesis measured by metabolic labeling was reduced in RPS19-depleted K562C and PC3 cells. To analyze the decrease in translational activity, we then used a classical assay to measure elongation rate (ribosome transit time) and employed a new method to measure ribosome run-off times after inhibition of translation initiation. The results indicate that, consistent with the reduction in protein synthesis, although polysome size is preserved, the induction of ribosomal stress in K562C cells caused a decrease in the average velocity of ribosomes in translating the mRNA, i.e. it impaired the rate of elongation. This finding was supported by the observation that the level of the inhibitory phosphorylation of eEF2 (Thr56) increased more than 2-fold in RPS19-depleted K562C cells and in PC3 cells after depletion of RPS6 or RPS7 or RPS19. The picture emerging from our analysis was, therefore, that the recruitment of TOP mRNAs in polysomes in response to ribosomal stress was not due to a specific stimulatory effect (e.g. on mTORC1) but rather to a general slowing down of translation elongation. In fact, it has been shown that mRNAs can be translated according to their capacity to compete for a limiting component of translation initiation (29,39), where ‘weak’ mRNAs compete inefficiently. Specific partial inhibition of elongation can make it, rather than initiation, the rate-limiting step of translation. Consequently, competition at the initiation stage is alleviated and the polysomal association of inefficient (‘weak’) mRNAs increases. Consistent with this model, treatment of K562C cells with a low dose of the elongation inhibitor cycloheximide also increased the percentage of TOP mRNAs associated with polysomes.

At this point, our data indicated that RPS19 depletion caused: (i) a 25–30% decrease of general protein synthesis, (ii) a 20–30% increase in the amount of TOP mRNAs associated to polysomes and (iii) a 50% decrease in elongation rate (in K562C cells). One interpretation of these findings was that the increase in polysome-associated TOP mRNAs could partly compensate the inhibition of elongation, allowing cells to preserve the synthesis of TOP mRNA-encoded proteins even when synthesis of other cellular proteins is impaired. To validate this hypothesis, we induced ribosomal stress in PC3 cells and measured (i) polysomal association of a well-established TOP mRNA (eEF1A) and a non-TOP mRNA (SOD) and (ii) the synthesis of the proteins encoded by such mRNAs. We found that the increased polysomal association of the eEF1A mRNA during RPS19 depletion did not lead to the synthesis of more eEF1A protein (which is observed following serum stimulation). However, increased polysomal association of its mRNA does ‘protect’ its synthesis from the inhibition observed for general protein synthesis and, e.g. for a protein (SOD) that is not encoded by a TOP mRNA.

Our data suggest that the inhibitory response to ribosomal stress at the level of protein synthesis is mediated by activation of eEF2K and phosphorylation of eEF2 on Thr56. eEF2K is a regulator of translation that has been shown to mediate cell survival in response to nutrient deprivation (40). The activity of eEF2K is inhibited by phosphorylation on Ser366 catalyzed by S6K1 and p90^RSK^ (35). We observed that ribosomal stress causes decreased phosphorylation of eEF2K at Ser366. This is consistent with the increase of phospho-eEF2 and the inhibition of translation elongation that we detected in our experimental conditions. The mTORC1 pathway, which by activating S6K1 would be a prime candidate for the regulation of eEF2K activity, did not show any change during ribosomal stress, similarly to what has been observed in HeLa cells after depletion of L7 (22). ERK phosphorylation is also unaffected, making unlikely a role of p90^RSK^ in eEF2K regulation.

In any case, we hypothesize that there is some kind of ‘sensor’ that ascertains whether the level of ribosomes is sufficient for the cellular needs and, in case of deficiency, would induce derepression of eEF2K and a consequent phosphorylation of eEF2. A potential candidate for this role is the mTORC2 complex, that has been recently shown to associate with ribosomes and to be involved in the response to ribosomal stress (22,41). One study (41) reported that inhibition of mTORC2 causes an increase of eEF2 phosphorylation, indicating that it may regulate either the kinase and/or phosphatase specific for this elongation factor. Another potential sensor of ribosomal stress is the protein kinase PIM1, which has been shown to interact with ribosomes and it is destabilized by ribosomal stress (42). The decrease in PIM1 level during ribosomal stress could cause, directly or indirectly, the activation of eEF2K. This possibility, however, remains to be addressed and other mechanisms could also be hypothesized.

**Figure 7:**
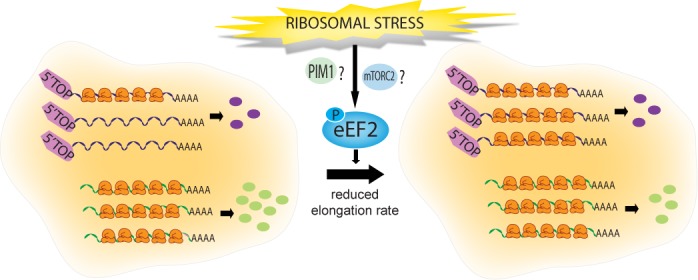
Model of TOP mRNA regulation in response to ribosomal stress. TOP mRNAs are poorly translated (compared to non-TOP mRNAs) and thus partly non-utilized because they are ‘weak’ competitors for translation initiation. In response to ribosomal stress, eEF2 is phosphorylated and the elongation rate is reduced. As a consequence, competition at elongation is reduced, and some of the unused TOP mRNAs are recruited into polysomes whereas more efficient non-TOP mRNAs cannot be recruited because they were already fully utilized. Therefore the synthesis of TOP mRNA-encoded proteins is maintained approximately constant (the recruitment roughly compensates the decrease of elongation rate) whereas the synthesis of other cellular proteins is reduced.

Independently of the specific mechanism, our data reveal a novel regulatory circuit that could be interpreted as a homeostatic control of ribosome biogenesis. In fact, as summarized in the model in Figure 7, ribosome deficiency activates a mechanism that, through inhibition of translation elongation, slows down the synthesis of all proteins except, e.g. RPs (whose mRNAs are recruited into polysomes allowing their synthesis to be maintained even though the overall elongation rate is slowed).

However, it may be that this regulation evolved simply to preserve the synthesis of crucial cellular components in critical growth conditions. Although a comprehensive survey of TOP mRNAs is not available, this group of messengers encodes proteins which are obviously essential for the cell, such as RPs and translation factors. The reason why other components of the translation machinery (e.g. aminoacyl-tRNA-synthetases, initiation factors) are not encoded by TOP mRNAs is not clear. The presence of the TOP structure in at the 5′-end of the mRNAs for all RPs (with no exceptions) and all translation elongation factors in vertebrates (43,44) indicates that it has an important regulatory function. It is intriguing that the synthesis of the main components of the translation apparatus (RPs and elongation factors) is regulated at the translational level. In addition, we report here that TOP mRNAs are specifically sensitive to alterations in the elongation rate. The fact that all elongation factors (but only a few among initiation and termination factors) are encoded by TOP mRNA strongly suggests the operation of a regulatory loop affecting elongation factor expression. Based on the present data, this could be an ‘autoregulatory loop’ whereby impaired elongation rates, which may result from inadequate levels of elongation factors, cause an increase in the translation of the mRNAs for elongation factors in order to rectify this insufficiency.

The translational reprogramming we observed following ribosomal stress could play a role in the pathological mechanism of Diamond-Blackfan Anemia (DBA). For instance, alteration of balance between proteins encoded by ‘weak’ and ‘strong’ mRNAs could affect hematopoiesis.

A number of studies indicate that a defect in ribosome synthesis causes the binding of some RPs to the ubiquitin ligase MDM2 leading to a stabilization of p53 (45,46). Which RPs are direct binders and therefore necessary for the stress response, it is still debated (47,48). In any case, our results may be consistent with this model. In fact, we find that ribosomal stress, through inhibition of translation elongation, favors the synthesis of all RPs including the putative binders to MDM2. It should be noted however that depletion of all RPs tested, both binders (RPL11, RPS7) and non-binders (RPS6, RPS19) caused a similar response at the level of TOP mRNA recruitment. In addition, p53-negative cells were also used in the experiments. Therefore the phenomenon we describe does not appear to be directly correlated with the RP-MDM2-p53 pathway.

It should be noted that our model suggests a rationale for the intrinsically low translational efficiency of mRNAs bearing the TOP sequence: it is a simple device to couple the synthesis of certain proteins required for adequate ribosome production with ribosomal stress. In this respect, the TOP sequence, similarly to some internal ribosomal entry sites (IREs)or upstream open reading frames (uORF) (49), represents a kind of *cis*-acting element necessary to preserve the translation of specific mRNAs in particular ‘emergency’ circumstances. An important goal for future work is the identification of the component(s) of the translational apparatus that act as the ‘selectivity factor’ that confers the selective control of 5′-TOP mRNAs by mTORC1 signaling. A good candidate would be the cap-binding protein eIF4E which, however, was seemingly excluded several years ago (50), but suggested again more recently (51). Similarly, the proposal that 4E-BP1 is a TOP mRNA regulator has been the subject of conflicting reports (6,7). In addition, the possibility of some kind of specific TOP mRNA binding factor has been investigated over many years without conclusive results (36). Therefore, the issue is still open with no clear hypothesis. Two general possibilities are: (i) the ‘selector’ is an unknown or uncharacterized component, such as, for instance, an isoform of an initiation factor (eIF4E-2, eIF4GII, etc.); (ii) more than one initiation factor participates in the ‘selection’ process, making their identification more difficult.

Finally, we think that the ‘weakness’ of TOP mRNAs, coupled with specific changes in the balance between initiation and elongation, could explain the ‘bimodal’ distribution of these mRNAs which was observed in the early studies: TOP mRNAs are either fully loaded onto polysomes or stored as subpolysomal messenger ribonucleoprotein (30,31). We think that the simultaneous modification of initiation and elongation rates could cause a change of the percentage of TOP mRNAs associated to polysomes without altering polysome size.

## SUPPLEMENTARY DATA

Supplementary Data are available at NAR Online.

SUPPLEMENTARY DATA
