# Supplementary material for: Ribosomal stress activates eEF2K–eEF2 pathway causing translation elongation inhibition and recruitment of Terminal Oligopyrimidine (TOP) mRNAs on polysomes
Source: Nucleic Acids Res. 2014 Oct 20;42(20):12668–80. doi: 10.1093/nar/gku996 (PMC4227798; doi:10.1093/nar/gku996)
Supplement: SUPPLEMENTARY DATA [file supp_42_20_12668__index.html]

Ribosomal stress activates eEF2K–eEF2 pathway causing translation elongation inhibition and recruitment of Terminal Oligopyrimidine (TOP) mRNAs on polysomes — SUPPLEMENTARY DATA 

# Ribosomal stress activates eEF2K–eEF2 pathway causing translation elongation inhibition and recruitment of Terminal Oligopyrimidine (TOP) mRNAs on polysomes

## SUPPLEMENTARY DATA

**Files in this Data Supplement:**

- SUPPLEMENTARY DATA
