# Supplementary material for: Ribosomal stress activates eEF2K–eEF2 pathway causing translation elongation inhibition and recruitment of Terminal Oligopyrimidine (TOP) mRNAs on polysomes
Source: Nucleic Acids Res. 2014 Oct 20;42(20):12668–80. doi: 10.1093/nar/gku996 (PMC4227798; doi:10.1093/nar/gku996)
Supplement: SUPPLEMENTARY DATA [file supp_gku996_nar-02147-a-2014-File009.zip › NAR-02147-A-2014 Suppl files/SupplementaryR1.docx]

**SUPPLEMENTARY MATERIAL**

**FIGURE LEGENDS**

**Figure S1. TOP mRNAs during ribosomal stress.** a) Cytoplasmic extracts from K562C cells untreated (-dox), K562 stably expressing control siRNA (+dox iCNT) or K562C cells treated for four days with doxycycline (+dox iS19) were separated on sucrose gradients. Fractions were collected and polysomal profiles were obtained while monitoring the optical density at 260nm (last panel). RNA extracted from the fractions was analyzed on Northern blots with probes for RPS19 (S19), RPS6 (S6), RPL7a (L7a) or β-actin (as control). Quantification is reported as bar graph of the percentage of mRNA associated to polysomes. Values represent the mean ± S.E. of at least three independent experiments. b) Total, nuclear or cytoplasmic RNA prepared from K562C untreated or treated with doxycycline was analyzed by Northern blot with the indicated probes.

**Figure S2. Ribosome amount in RP depleted cells.** a) Cytoplasmic extracts from K562C cells untreated (-dox) or treated for four days with doxycycline (+dox) were separated on sucrose gradients. Profiles were obtained by collecting gradient fractions while monitoring the optical density at 260nm. b) Cytoplasmic extracts from PC3 cells transfected with a control siRNA (siCNT) or siRNAs against RPS6 (siS6) or RPL11 (siL11) were analyzed as in (a). c) Cytoplasmic extracts from PC3 cells transfected with a control siRNA (siCNT) or siRNAs against RPS19 (siS19) were analyzed by western blot with specific antibodies (RPS19, GAPDH) and by northern blot with probes specific for 18S after sucrose gradient fractiionation. Total protein amount was estimated with the bradford assay. Western and northern blots were quantified as described in the previous figures.

**Figure S3. Scheme of transit time experiment.** After 1 transit time the nascent chains are completely labeled and an equal amount of radioactivity will be in the released peptides. After the second transit time the radioactivity in the nascent peptides will remain constant whereas the labeling in the released chains will be double. In each following transit time an amount of radioactivity as in the second transit time will be released. The graph of the released radioactivity as a function of time is linear after one transit time. The intercept on the x-axis of this plot indicates the time at which radioactivity appeared in the released chains. The graph of total radioactivity (which includes nascent plus released polypeptides) is also linear after one transit time. The intercepts on the x-axis indicates the time at which incorporation began. The difference in the intercepts is the time that the “average” ribosome takes to complete the scanning of the mRNA (and release the polypeptide). This time is defined “half transit time” (time necessary to scan half of the mRNA) because the average ribosome can be assumed to be in the middle of an mRNA.

**Figure S4. Analysis of translation elongation.** Cytoplasmic extracts from PC3 cells transfected with a control siRNA (siCNT) or with siRNA against RPS19 (siS19) untreated (T=0’) or treated for 5, 7.5 and 10 minutes with harringtonine (T=5’, T=7.5, T=10’) were separated on sucrose gradients and analyzed as in Fig. 1.
